# Differences in Tissue and Species Tropism of Reptarenavirus Species Studied by Vesicular Stomatitis Virus Pseudotypes

**DOI:** 10.3390/v12040395

**Published:** 2020-04-02

**Authors:** Yegor Korzyukov, Rommel Iheozor-Ejiofor, Lev Levanov, Teemu Smura, Udo Hetzel, Leonora Szirovicza, Juan Carlos de la Torre, Luis Martinez-Sobrido, Anja Kipar, Olli Vapalahti, Jussi Hepojoki

**Affiliations:** 1 Medicum, Department of Virology, University of Helsinki, 00100 Helsinki, Finland; yegor.korzyukov@helsinki.fi (Y.K.); rommel.iheozor-ejiofor@helsinki.fi (R.I.-E.); lev.levanov@helsinki.fi (L.L.); teemu.smura@helsinki.fi (T.S.); leonora.szirovicza@helsinki.fi (L.S.); olli.vapalahti@helsinki.fi (O.V.); 2 Vetsuisse Faculty, Institute of Veterinary Pathology, University of Zürich, 8006 Zürich, Switzerland; udo.hetzel@uzh.ch (U.H.); anja.kipar@uzh.ch (A.K.); 3 Department of Veterinary Biosciences, Faculty of Veterinary Medicine, University of Helsinki, 00100 Helsinki, Finland; 4 Department of Immunology and Microbial Science IMM-6, The Scripps Research Institute, La Jolla, CA 92037, USA; juanct@scripps.edu; 5 Texas Biomedical Research Institute, San Antonio, TX 78227, USA; 6 Department of Virology, Helsinki University Hospital, University of Helsinki, 00100 Helsinki, Finland

**Keywords:** BIBD, reptarenavirus, arenavirus, VSV, pseudotype, tissue tropism

## Abstract

Reptarenaviruses cause Boid Inclusion Body Disease (BIBD), and co-infections by several reptarenaviruses are common in affected snakes. Reptarenaviruses have only been found in captive snakes, and their reservoir hosts remain unknown. In affected animals, reptarenaviruses appear to replicate in most cell types, but their complete host range, as well as tissue and cell tropism are unknown. As with other enveloped viruses, the glycoproteins (GPs) present on the virion’s surface mediate reptarenavirus cell entry, and therefore, the GPs play a critical role in the virus cell and tissue tropism. Herein, we employed single cycle replication, GP deficient, recombinant vesicular stomatitis virus (VSV) expressing the enhanced green fluorescent protein (scrVSV∆G-eGFP) pseudotyped with different reptarenavirus GPs to study the virus cell tropism. We found that scrVSV∆G-eGFPs pseudotyped with reptarenavirus GPs readily entered mammalian cell lines, and some mammalian cell lines exhibited higher, compared to snake cell lines, susceptibility to reptarenavirus GP-mediated infection. Mammarenavirus GPs used as controls also mediated efficient entry into several snake cell lines. Our results confirm an important role of the virus surface GP in reptarenavirus cell tropism and that mamma-and reptarenaviruses exhibit high cross-species transmission potential.

## 1. Introduction

Viral zoonoses can have severe consequences on human health as illustrated by viral hemorrhagic fevers (VHFs) caused by filo-, flavi-, hanta-, nairo-, and arenaviruses [1,2,3,4,5,6,7]. These viruses cause long-term, usually subclinical, chronic infections in their natural host reservoirs [8]. However, upon zoonotic transmission, these viruses can cause severe disease in humans via different mechanisms [4,9,10].

Boid Inclusion Body Disease (BIBD), initially recognized in the 1970s, affects mainly captive boas and pythons [11]. BIBD is characterized by the formation of electron dense intracytoplasmic inclusion bodies (IB) [12] in various cell types (including blood cells) and tissues [13,14,15]. BIBD spreads efficiently within collections, and the recommendation to euthanize snakes with BIBD can lead to loss of entire collections [11]. Clinical manifestations of BIBD are variable and include central nervous system (CNS) signs such as head tremors, loss of coordination, and regurgitation [11]. Death due to secondary bacterial, fungal or protozoal infections and neoplastic diseases is common in snakes with BIBD [16], suggesting that BIBD might be associated with an impaired immune response.

The causative agent for BIBD remained unknown until the early 2010s, when novel arenaviruses were identified in snakes with BIBD [13,15,17], which extended arenaviruses host range to species other than the well-established rodent reservoirs [1,2,3]. These findings led to establishment of two genera, *Mammarenavirus* and *Reptarenavirus*, within the family *Arenaviridae* [18,19]. We and others have documented that snakes with BIBD are often co-infected with several reptarenaviruses [20,21], and we discovered another novel arenavirus in snakes, Haartman Institute Snake virus-1 (HISV-1) [20], which later became the type species of a third arenavirus genus, *Hartmanivirus* [19,22]. The finding that IBs contain reptarenavirus NP further supported the link between reptarenavirus infection and BIBD [15]. Experimental infection of boas and pythons with purified reptarenaviruses provided unequivocal evidence that reptarenaviruses can cause BIBD [14]. Interestingly, in this experiment, pythons developed strong CNS signs without evidence of IB formation, whereas seemingly healthy boas harbored large IBs in most tissues [14]. Moreover, we have documented vertical transmission of reptarenaviruses and hartmaniviruses in boas, and the swarm of reptarenavirus genome segments transmitted can vary between the offspring [23]. The origins and reservoir hosts of both reptarenaviruses and hartmaniviruses remain unknown, as the viruses have only been found in captive snakes [13,15,17,24,25].

Arenaviruses have a negative-sense bi-segmented RNA genome, with the exception of members of the most recently recognized arenavirus genus, *Antennavirus*, identified in fish, which have a tri-segmented genome [19]. The large (L) genome segment encodes the RNA-dependent-RNA-polymerase (RdRp) and the matrix-like Z protein (ZP) [26,27], but hartmaniviruses lack the ZP [22]. The small (S) segment encodes the glycoprotein precursor protein (GPC) and the NP [28,29,30,31]. The mammarenavirus GPC is initially cleaved by cellular signal peptidase [32] and later by subtilisin kexin isozyme-1 (SKI-1)/site-1 protease (S1P) producing a stable signal peptide (SSP) and glycoproteins GP1 and GP2 [33]. While reptarenavirus GPCs appear to lack the SSP, it exists in hartmanivirus GPCs [22]. Furin, instead of SKI-1/S1P host cell protease, presumably mediates the secondary processing of hartmanivirus GPC [22]. Trimers of the SSP-GP1-GP2 complex comprise the virion spike of mammarenaviruses [34]. GP1 mediates binding to the cell surface receptor [35,36,37,38], and GP2 directs the fusion with endosomal membranes [39,40]. SSP also participates in the fusion process [41,42].

GP1 binding to the cell surface receptor represents the first step of arenavirus entry into a new host cell [35,38]. Accordingly, cell surface receptor expression pattern contributes to arenavirus cell and tissue tropism [43]. The conserved and widely expressed receptor for extracellular matrix proteins, α-dystroglycan (α-DG), is a main receptor for Old World (OW) mammarenaviruses, such as lymphocytic choriomeningitis virus (LCMV) and Lassa virus (LASV) [38]. Secondary alternative receptors, including members of the Tyro3/Axl/Mer and T-cell immunoglobulin mucin (TIM) receptor families may account for LASV and LCMV infection of cells lacking fully glycosylated α-DG [44,45]. Cell entry of the OW hemorrhagic fever (HF) mammarenavirus Lujo virus (LUJV) is mediated by neuropilin (NRP)-2, a cell-surface receptor for semaphorins [46]. NRP-2 is highly expressed in microvascular endothelial cells, which may contribute to LUJV-induced coagulopathy. The completion of the cell entry process for LASV and LUJV involves a late endosomal receptor switch mechanism, the late endosomal resident proteins LAMP and CD36 for LASV and LUJV, respectively [46,47]. Human transferrin receptor 1 (TfR1) is the main cellular receptor used for cell entry of pathogenic New World (NW) mammarenaviruses, including Junin virus (JUNV) and Machupo virus (MACV) [35].

Hartmaniviruses appear to target neuronal and smooth muscle cells [22], while reptarenaviruses infect a wide range of cell types [14,48]. Interestingly, snakes with BIBD often carry more L than S segments [20,21,23], and the distribution of segments may vary between tissues of the same individual [20,23]. The difficulties in obtaining isolated L and S segment pairs significantly hampers the studies on the role of the GPs in reptarena-and hartmanivirus cell entry.

Here, we document the use of single cycle replication, GP deficient, recombinant vesicular stomatitis virus (VSV) expressing the enhanced green fluorescent protein (scrVSVΔG-eGFP) pseudotyped with arenavirus GPs to study reptarenavirus GP-mediated cell tropism. Our results show that reptarenavirus GPs efficiently provide entry into a wide range of mammalian cell lines, and correspondingly mammarenavirus GPs enable entry into culture reptilian cell lines, suggesting that arenaviruses could possess a high cross-species transmission potential.

## 2. Materials and Methods

### 2.1. Cell Lines

We made use of the following mammalian cell lines: African green monkey kidney (Vero E6, American Type Culture Collection, ATCC), human lung carcinoma (A549, ATCC), human embryonic kidney (HEK293FT, Thermo Fisher Scientific, Waltham, MA, USA), human neuroblastoma (SK-N-SH, ATCC), Chinese hamster ovary (CHO, ATCC), and baby hamster kidney (BHK-21, ATCC) cells. We maintained the mammalian cells at standard conditions (5% CO_2_, 37 °C) in medium supplemented with 10% fetal bovine serum (FBS), 2 mM L-glutamine, 100 IU/mL penicillin, and 100 μg/mL streptomycin. We used minimal essential medium (MEM) for Vero E6 and A549, and Dulbecco’s Modified Eagle Medium (DMEM) for the other cell lines. In addition, we used the following reptilian cell lines: *Boa constrictor* kidney (I/1Ki, in MEM), lung (V/5Lu, DMEM), liver (V/1Liv), heart (V/2Hz, DMEM), and brain (V/4Br, DMEM) cells, and *Morelia viridis* liver (VII/2Liv, DMEM) cells described in [15,49,50]. We maintained the snake cells at 5% CO_2_ and 30 °C, and used collagen-coated (as described [50]) bottles and plates for VII/2Liv and V/1Liv cells.

### 2.2. Phylogenetic Analysis

We retrieved the arenavirus amino acid sequences from GenBank and aligned them using INS-i algorithm embedded in MAFFT version 7 [51]. The phylogenetic tree was inferred using the Bayesian method implemented in MrBayes v3.1.2 [52] and the Blosum model of amino acid substitution. MrBayes was run for a million generations and sampled every 5000 generations, with final standard deviation of 0.005 between 2 runs.

### 2.3. Plasmids and Cloning

We selected the following arenavirus GPCs to be cloned into the pCAGGS-HA (pCAGGS-FLAG for aurora borealis virus-1, ABV-1) vector [53]: University of Helsinki virus-1 (UHV-1, GenBank accession no. KR870011.1), UHV-2 (KR870016.1), University of Giessen virus-1 (UGV-1, NC_039005.1), ABV-1 (KR870010.1), ABV-2 (KR870018.1), Golden Gate virus-1 (GGV-1, NC_018483.1), CAS virus-1 (CASV-1, JQ717262.1), tavallinen suomalainen mies virus-2 (TSMV-2, KX527575), GPC from S5 segment (S5-like, KX527579.1), Haartman Institute snake virus-1 (HISV, NC_043444.1), JUNV (NC_005081.1), and LCMV (AY847350.1). The UHV-2, UGV-1, HISV-1, LCMV, and JUNV GPC constructs are described in [50]. We used the following primers for RT-PCR amplification of ABV-1, ABV-2, TSMV-2, and S5-like GPCs: ABV-1_FWD 5’-TTATGAGCTCATGGCGGGTCAGACTC-3´, ABV-1_REV 5´-ATAACCCGGGCCTTCTCACCCAGC-3´, ABV-2_FWD 5´-TTATGAGCTCATGGCAGGCTGGGC-3´, ABV-2_REV 5´-ATAAATGCATTCTTCTAACCCAACTGCACAC-3´, TSMV-2_FWD 5´-TTATGAGCTCATGGCGGGCTGGAT-3´, TSMV-2_REV 5´-ATAACCCGGGCCTTCTCACCCAACTACAC-3´, S5_FWD 5´-TTATGAATTCATGGCACCCACTCTGATG-3´, S5_REV 5´-ATAACCCGGGTCTCTTGACCCAGCT -3´. The RT-PCR products were cloned into pCAGGS-HA expression plasmid as described [50]. We ordered the GPCs of CASV-1 and GGV-1 GPCs as synthetic genes from GeneUniversal (Newark DE USA), subcloned them into pCAGGS-HA and produced plasmid maxipreps as described [50]. We verified the inserts by Sanger sequencing (DNA Sequencing and Genomics Laboratory, Institute of Biotechnology, University of Helsinki).

### 2.4. Expression of Arenavirus GPCs, Cell Surface Biotinylation, and Purification of Biotinylated Proteins

HEK293FT cells served to produce the pseudotypes bearing arenavirus GPs. We transfected HEK293FT cells using FuGENE HD (Promega, Madison, WI, USA), plated the HEK293FT cells onto 12-well plates, allowed them to reach ~80% confluency, and replaced the medium (as above but with 5% FBS and without antibiotics) on the day of transfection. We prepared the reagent:DNA mixes (4:1 ratio) by diluting the plasmid into 51 μL of OptiMEM (Sigma, Saint Louis, MO, USA) to yield 0.02 μg/μL, added 4.4 μL of Fugene HD, mixed by pipetting, allowed the complexes to form for 15 min at room temperature (RT), added the mix onto the cell layers, and incubated the cells for 48 h.

To analyze the processing and trafficking of the expressed GPs to the cell surface, we washed the transfected cells at 48 h post transfection three times with phosphate-buffered saline (PBS), and treated the cells with EZ-Link Sulfo-NHS-SS-Biotin (ThermoFisher Scientific, Waltham, MA, USA) following the manufacturer’s protocol. After the labelling reaction, we washed the cells three times with Tris-buffered saline (TBS, pH 7.4), lysed the cells in 50 mM Tris, 150 mM NaCl, 1% Triton X-100, pH 8.0, supplemented with EDTA-free protease inhibitor cocktail (Roche, Basel, Switzerland), and cleared the lysate by centrifugation (5 min, 10,000× *g*). Pierce Monomeric Avidin Agarose (ThermoFisher Scientifc, Waltham, MA, USA), used according to the manufacturer’s protocol, served to pull down the biotinylated proteins. We eluted the bound protein to Laemmli sample buffer by boiling, and analyzed the proteins as described under immunoblotting.

### 2.5. Generation of VSV Pseudotyped with Arenavirus GPs

To generate VSV pseudotypes, we inoculated the transfected cells at 48 h post transfection with rVSV-ΔG stock prepared as described [54]. Briefly, we added 300 μL/well (corresponding to a multiplicity of infection of 3 to 5) of rVSV-ΔG, incubated the cells for 1–1.5 h on an orbital shaker at RT, removed the inoculum, added fresh medium (as above but with 5% FBS and without antibiotics), and incubated the cells for 48 h. After 48 h infection (and 96 h after transfection), we collected the cell culture supernatants, filtered them through a 0.45 µm filter (Millipore, Burlington, MA, USA), and pelleted the viruses by ultracentrifugation (Beckman coulter [Brea, CA, USA] SW-55 rotor, 50,000× *g*, 4 °C, 1 h) using a 0.5 mL 20% (*w*/*v*) sucrose cushion. Pelleted viruses were resuspended in PBS by gentle pipetting, and the aliquots were stored at 4 °C or −80 °C for further use.

### 2.6. Infections with Pseudotyped Viruses

We titrated the pseudovirus stocks and pelleted virions using V/2Hz cells grown on 96-well plates. Briefly, we prepared a 10-fold dilution series (from 1:10 to 1:10^8^) for each stock, added 10 μL of diluted virus onto cells grown on 96-well plates, incubated (5% CO_2_, 30 °C) the plates for 48–60 h, and enumerated the fluorescent focus forming units per ml (FFFUs/mL) using fluorescence microscopy.

For studying the cell tropism of the pseudotyped viruses in mammalian and reptilian cells, we seeded the different cell lines onto 96-well plates and inoculated the cells with the pseudotyped viruses diluted according to the initial titration on V/2Hz cells, aiming at approximately 250–500 FFFUs per well. We removed the scrVSVΔG-eGFP pseudovirus inocula after 2 h incubation, supplied fresh medium, and incubated the cells (snake cells at 5% CO_2_ and 30 °C; mammalian cells at 5% CO_2_ and 37 °C) for 48–60 h. After the incubation, we fixed the cells with 4% paraformaldehyde (PFA; pH 7.4) and stained the nuclei with Hoechst 33342. We used quadruplicates for each pseudotype, and scrVSVΔG-eGFPs pseudotyped with VSV G and naked scrVSVΔG-eGFPs (i.e., the supernatant collected from non-transfected cells inoculated with scrVSVΔG-eGFP pseudotyped with VSV G). The Opera Phenix High Content Screening System (PerkinElmer, Waltham, MA, USA), provided by FIMM (Institute for Molecular Medicine Finland, High Content Imaging and Analysis [FIMM-HCA]), served to enumerate the infected cells.

### 2.7. Immunoblotting

The bicinchoninic acid (BCA) protein assay kit (Pierce, Thermo Scientific, Waltham, MA, USA) was employed to determine the protein concentrations of the cell lysates in 50 mM Tris, 150 mM NaCl, 1% Triton X-100, pH 8.0, supplemented with EDTA-free protease inhibitor cocktail (Roche, Basel, Switzerland). We used ready-made 4–20% Mini-PROTEAN^®^ TGX (Bio-Rad, Hercules, CA, USA) to separate the proteins (5 μg of total protein per lane for the lysates) and wet blotting to transfer the proteins onto nitrocellulose membranes (GE Healthcare, Chicago, IL, USA) according to standard protocols (https://www.gelifesciences.com/en/us/solutions/protein-research/knowledge-center/western-blotting/comparison-of-transfer-methods-in-western-blotting) as described [55]. After 30 min blocking in 50 mM Tris, 150 mM NaCl, 0.05% Tween 20, pH 7.4 (TBS-T) supplemented with 3% skimmed milk and 1% BSA at RT on an orbital shaker, the membranes were incubated with the primary antibodies (mouse anti-HA-tag [clone 16B11, BioLegend, San Diego, CA, USA] at 1:1000 and mouse anti-VSV-M [clone 23H12, KeraFast, Boston, MA, USA] at 1:2000 dilution in blocking buffer) overnight at 4 °C, washed three times with TBS-T, probed with the secondary antibody (donkey anti-mouse AlexaFluor800 at 1:10,000 dilution in blocking buffer) for 1 h at RT, washed three times with TBS-T and twice with TBS, and recorded the results with the Odyssey Infrared Imaging System (LI-COR biosciences, Lincoln, NE, USA).

## 3. Results

### 3.1. Production of Arenavirus GP-Pseudotyped scrVSV∆G-eGFPs in HEK293FT Cells

**Figure 1:**
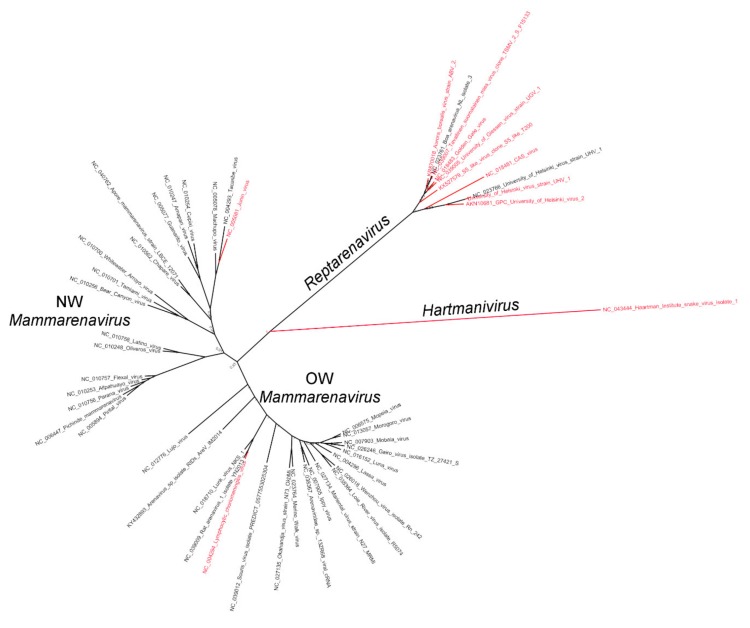
A phylogenetic tree based on the amino acid sequences of arenavirus GPCs. The GPCs included in the study are in red font.

Snakes with BIBD often harbor more than one S segment [20,21], and an infected snake can carry differing sets of L and S segments in different tissues [20]. To examine whether, as with mammarenavruses, reptarenavirus GPC gene defined cell type and species tropism, we generated pseudotyped forms of the scrVSVΔG-eGFP bearing different reptarenavirus GPs. We selected the GPs based on phylogenetic analysis (Figure 1) and included representatives of OW (LCMV) and NW (JUNV) mammarenaviruses and hartmanivirus (HISV-1) as controls.

**Figure 2:**
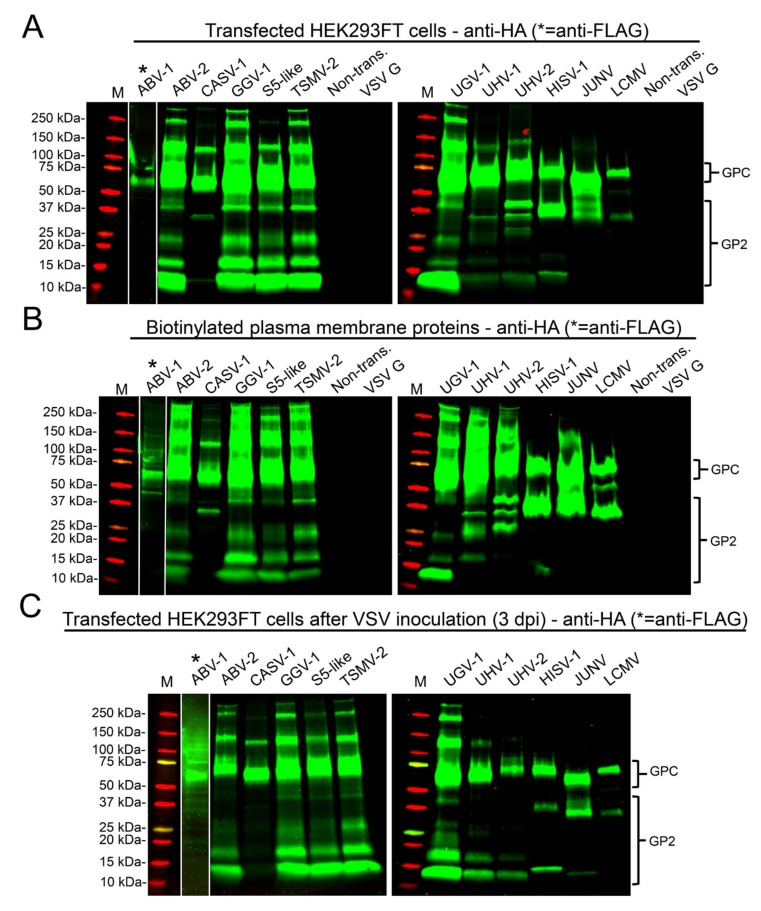
Immunoblots of transfected cells and concentrated VSV pseudotypes. (**A**) Expression of arenavirus GPCs in HEK293FT. The proteins separated on SDS-PAGE and transferred onto nitrocellulose were probed with an anti-HA antibody. (**B**) Anti-HA immunoblot of biotinylated plasma membrane proteins from cells transfected with arenavirus GPCs. (**C**) Anti-HA immunoblot of HEK293FT cells transfected with arenavirus GPCs at 3 days post VSV inoculation. In A to C, the intact GPCs migrate at approximately 60–75 kDa and the cleaved GP2s between 10 and 37 kDa, as indicated. Anti-FLAG antibody served for ABV-1 GPC detection from the same membrane, but due to differences in the band intensity the anti-FLAG staining is shown as a strip overlaid on top of the anti-HA staining. M = molecular weight marker (Precision Plus Protein Dual Color Standards, Bio-Rad). Odyssey Infrared Imaging System (LI-COR) served to record the results.

To assess the expression and processing of reptarenavirus GPCs occurs in mammalian cells, we transfected HEK293FT cells with plasmids expressing HA-epitope tagged forms of arenavirus GPCs and assessed their expression by immunoblotting. Reptarenavirus GPCs efficiently expressed in mammalian cells but processing of GPCs was not very efficient (Figure 2). The observed size variation between the different GPCs likely reflects differences in both their length and glycosylation. The HA-tag was located in the C-terminus of GPC and therefore it detected GP2. Reptarenavirus GP2s have a consistent size of approximately 190 amino acids, slightly smaller than GP2s of mammarenaviruses and hartmaniviruses [22]. Among the multiple mono- and polyclonal anti-HA antibodies we tested, all detected GPC, but only one detected processed GPC (i.e., GP2). Processing of reptarenavirus GPCs produced multiple bands migrating between the 10 and 37 kDa marker lanes (Figure 2A). Bands migrating between 25 and 37 kDa marker likely represent GP2 species with different degree of glycosylation. Bands migrating at 10 and 15 kDa may represent products of proteolytic degradation of the GP2, or polypeptides produced via alternative transcription or translation initiation, or from leaky scanning. GPCs of hartmani- and mammarenavirus were expressed and processed, as judged by bands migrating close to the 37 kDa marker, likely corresponding to GP2.

To determine whether the GPCs and processed GP2s trafficked to the plasma membrane in HEK293FT cells, we compared the migration pattern of cell surface biotinylated proteins and whole cell lysates in cells transfected with arenavirus GPCs. Immunoblots of cell surface biotinylated proteins (Figure 2B) showed a similar pattern to that of whole cell lysates (Figure 2A), indicating efficient transport of the expressed proteins to the plasma membrane. We observed the same protein expression pattern in HEK293FT cells transfected with all the plasmids required for production of pseudotyped scrVSVΔG-eGFP (Figure 2C).

We tested the infectivity of pseudotyped scrVSVΔG-eGFP on the boid heart cell line (V/2Hz), and compared their infectivity to that of the positive, scrVSVΔG-eGFP pseudotyped with VSV G, and negative, scrVSVΔG-eGFP, controls. The supernatants collected from cells transfected with arenavirus GPCs containing the pseudotyped scrVSVΔG-eGFP particles were used in subsequent experiments, further demonstrating that the GPC processing produced particles capable of entering different cell lines.

### 3.2. Differences in Tissue and Species Tropism of Reptarenaviruses

**Figure 3:**
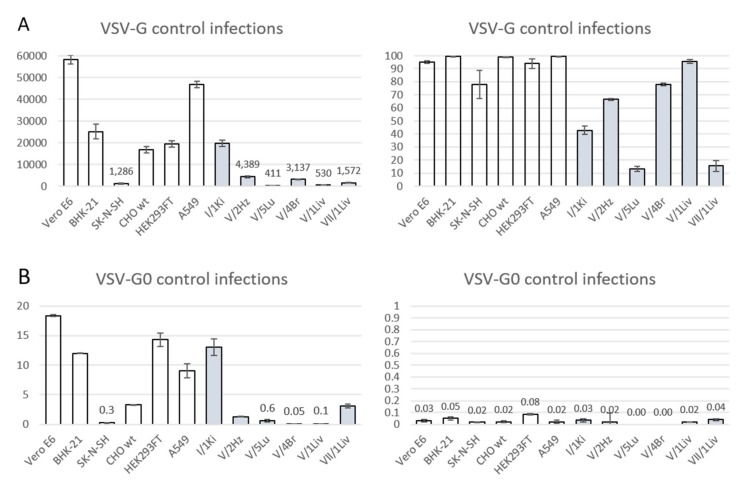
Control infections on all cell lines with naked VSV (VSV-G0) and VSV pseudotyped with its own GP (VSV-G). The X-axes represent the different cells lines and the Y-axis the number of infected cells (left) or the percentage of cells infected (right). The values represent the average of four measurements, and the error bars show standard deviation. White bars represent mammalian and grey bars reptilian cell lines. (**A**) Infection efficiency of VSV-G in mammalian (white bars) and reptilian (grey bars) cell lines. (**B**) Infection efficiency of VSV-G0 in mammalian (white bars) and reptilian (grey bars) cell lines. Opera Phenix high content image screening system served to quantify the infected cells based on eGFP expression and staining of nuclei with Hoechst 33342.

We used scrVSVΔG-eGFP pseudotyped with nine reptarena-, one hartmani-, and two mammarenavirus GPCs to infect a panel of six mammalian and six reptilian cell lines, and used scrVSVΔG-eGFP pseudotyped with VSV-G (scrVSVΔG-eGFP/VSV-G) as positive control. scrVSVΔG-eGFP pseudotyped with GPCs of JUNV and LCMV, with known receptors [35,38,56,57], served as additional controls to assess GP-mediated cell entry into mammalian cells. scrVSVΔG-eGFP/VSV-G efficiently infected efficiently all mammalian cell lines we tested, ranging from 70% (SK-N-SH) to 97% (BHK-21). We observed a greater variation in the ability of scrVSVΔG-eGFP/VSV-G to infect reptilian cells, 12% (V/5Lu, boa lung) to 85% (V/1Liv, boa liver (Figure 3A). The negative control scrVSVΔG-eGFP infected less than 0.1% of cells in all cell lines tested (Figure 3B).

**Figure 4:**
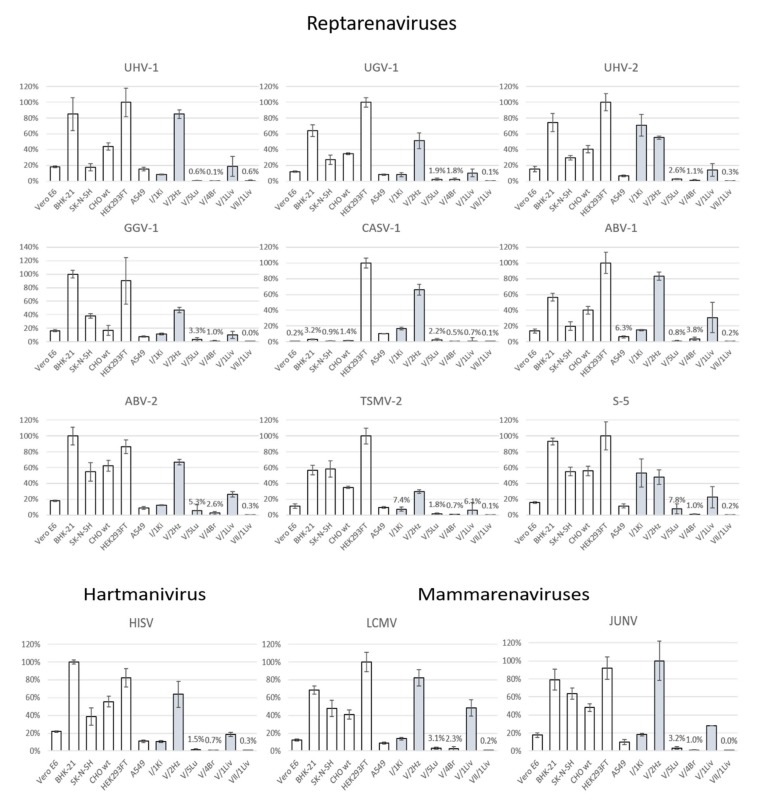
Infectivity of cell lines by pseudotyped viruses, indicating cell count of GFP positive cells. The mammalian cell lines are presented by white bars and the reptilian cell lines by grey bars, the X-axes indicate the individual cell lines and the Y-axes show the permissiveness in relation to VSV-G pseudotypes, expressed as relative percentage in comparison to the most permissive cell line. The values represent the average of four measurements, and the error bars show standard deviation. Abbreviations for the viruses: UHV-1—University of Helsinki Virus-1, UHV-2—University of Helsinki Virus-2, UGV-1—University of Giessen virus-1, GGV-1—Golden Gate Virus-1, CASV-1—California Academy of Sciences virus-1, ABV-1—auroa borealis virus-1, ABV-2—aurora borealis virus-2, TSMV-2—tavallinen suomalainen mies virus-2, S-5—S-5 like segment virus, HISV-1—Haartman Institute snake virus-1, LCMV—Lymphocytic choriomeningitis virus, JUNV—Junin virus.

We calculated the infectivity of each pseudotyped scrVSVΔG-eGFP produced on each cell line based on the dilution used and the number of fluorescent foci (Table 1), which revealed differences among reptarenavirus GPs in their ability to mediate cell entry. To avoid the confounding factor related to differences in VSV replication and gene expression among the different cell types, we normalized the infectivity values for VSVs pseudotyped with arenavirus GPs to those obtained with scrVSVΔG-eGFP/VSV-G in each cell line (Figure 4).

All the tested arenavirus GPs provided efficient entry into mammalian cells, whereas only JUNV GP showed preference to a reptilian cell line (V/2Hz, boa heart). Of the reptarenavirus GPs, the one of CASV-1 very efficiently mediated entry into HEK293FT and V/2Hz cells, whereas other cell lines were less susceptible to CASV-1 GP-mediated cell entry (Table 1). The tested arenavirus GPs showed low variation in their ability to provide entry into Vero E6 cells, commonly utilized for virus propagation. BHK-21 cells were highly permissive to cell entry mediated by GPs of all arenavirus tested except for that of CASV-1. Similarly, all tested arenavirus GPs provided efficient entry into the SK-N-SH, CHO and HEK293FT cell lines. A549 cells were also permissive to arenavirus GP bearing pseudotypes.

Intriguingly, only two, UHV-2 and S5, of the reptarenavirus GPs efficiently facilitated entry into I/1Ki, the boa kidney cell line employed in our earlier studies [15,22,48,50], indicating that factors other than entry contribute to reptarenavirus cell and tissue tropism. All tested arenavirus GPs, including those of hartmani- and mammarenaviruses, efficiently mediated entry into the V/2Hz cell line. The V/5Lu cell line showed low permissiveness for most arenavirus GPs. The low infectivity of scrVSVΔG-eGFP/VSV-G in V/5Lu cells (Figure 3) may account for the observed results, suggesting restricted VSV replication and gene expression in this cell line. V/4Br cells exhibited low permissiveness for all tested arenavirus GPs. Most arenavirus GPs, except CASV-1 and TSMV-2, mediated cell entry into V/1Liv cells at moderate efficiency, a finding consistent with the observation that hepatocytes consistently exhibit IB in snakes with BIBD. GPs of UHV-1, UHV-2, ABV-1, and ABV-2 provided low entry efficiency into python liver cells (VII/1Liv, green tree python). The inability of VII/1Liv cells to support efficient VSV replication, as suggested by the low number of infected cells observed with scrVSVΔG-eGFP/VSV-G control (Figure 3), could partially explain this result.

## 4. Discussion

Arenavirus infection at the cellular level is initiated by the interaction of the virus surface GP with its receptor on the cell surface. A main cellular receptor for LCMV, LASV and several other OW mammarenaviruses is alpha dystroglycan (α-DG) [38,56], whereas pathogenic NW mammarenaviruses use the human transferrin receptor-1 (TfR1) for their entry [35], and LUJV, which does not cluster with either NW or OW mammarenaviruses, uses neuropilin-2 (NRP2) [46]. The cellular receptors used by reptarenaviruses and hartmaniviruses are unknown. Reptarenaviruses, and occasionally hartmaniviruses, are found in snakes with BIBD, and co-infections by multiple reptarenaviruses appear common [20,21]. Evidence suggest that reptarenaviruses have a very broad tissue tropism, which indicates that they use a ubiquitously expressed receptor. However, these studies only allowed identification of the viruses at genus level [13,15]. Our recent data suggest that there is variation in the distribution of L and S segments between different tissues of an individual infected snake [23]. Herein, we used pseudotyped rscVSVΔG-eGFP to characterize the ability of reptarenavirus GPs to mediate cell entry into a variety of mammalian and reptilian cell lines. Our study included the GPs of nine reptarenaviruses and one hartmanivirus (HISV-1), the GPs of two mammarenaviruses (LCMV and JUNV) served as controls with known receptor usage (Figure 1). We used rscVSVΔG-eGFP and rscVSVΔG-eGFP/VSV-G as controls to evaluate the assay background and the ability of VSV to replicate in different cell lines.

Mammarenavirus GPC processing into SSP, GP1 and GP2 is required for the GP2-mediated pH-dependent fusion event required to release the viral ribonucleoprotein (vRNP) into the cytoplasm of the host cell, where vRNP directs replication and transcription of the viral genome [58,59,60]. Therefore, we evaluated processing of various arenavirus GPCs in mammalian HEK293FT cell line. We used C-terminal HA-tagged GPCs to facilitate detection of GPC and GP2. We tested several poly- and monoclonal anti-HA antibodies, and found that only one of them efficiently detected both GPC and GP2. We observed processing of all GPCs tested in HEK293FT, but at relative low efficiency (Figure 2A). Cells transfected with reptarenavirus GPCs showed often four bands with varying intensity around the 10–37 kDa region (Figure 2A), with bands migrating at approximately 25 and 37 kDa likely representing GP2. The prominent bands at approximately 10 and 15 kDa may correspond to GP2 degradation fragments, or products from leaky ribosome scanning or alternate translation initiation sites. The results from biotinylation studies indicate that the expressed GPs are efficiently trafficked to the plasma membrane (Figure 2B). The migration pattern of biotinylated proteins looked similar to that observed when analyzing proteins in whole cell lysates, indicating that unprocessed GPCs reached the plasma membrane. We confirmed the same pattern of GPC expression and processing in cells transfected with all the plasmids required for production of pseudotyped rscVSVΔG-eGFP (Figure 2C), but the amount of GP2s appeared to be lower (when comparing the intensities of the GPC and GP2 bands) under these conditions. We also tested the ability of I/1Ki (boid kidney) cells to mediate production of VSV pseudotyped with arenavirus GPs and found that the particles produced in both HEK293FT and I/1Ki had a similar ability to infect mammalian and reptilian cell lines.

Most of our primary cell lines originated from euthanized juvenile three to 21 day-old Boa constrictor imperator snakes. We noted a variety of cell subpopulations as judged by cell pleomorphism in the initial passages of the cell lines (up to 15 passages). At later passages (from 15 to 30) the cultures begun to demonstrate monomorphous cells, which we interpret as a narrow population of stable cell lines (ages from 5 to 15 years, and at passages 45–130) derived from the particular organ. According to our immunohistological analyses we characterize them as follows: I/1KI, epithelial (tubular) cells; V/2Hz, cardiomyocytes; V/5Lu, (vascular) smooth muscle cells; V/4Br, (vascular) smooth muscle cells; and V/1Liv, hepatic stellate cells; the VII/1Liv remains uncharacterized. The ability of different reptarenavirus GPs to mediate entry into reptilian cells did not vary considerably (Figure 4 and Table 1), with the exception of the CASV-1 GP that showed much higher efficiency in HEK293FT and V/2Hz cell lines. These differences could reflect that CASV-1 originates from a *Corallus annulatus* [13], whereas other viruses infect boa constrictors. UHV-2 and S5-like GPs showed the highest entry efficiency in I/1Ki cells, the cell line which we routinely employ for the isolation of reptarenaviruses [15,20,22,23,48]. Interestingly, HISV-1 and UHV-2, originally isolated together [20,22], showed a similar preference for mammalian cell lines but significantly differed in their ability to infect the boid kidney (I/1Ki) cells. The low entry efficiency of pseudotypes with reptarenavirus GPs into I/1Ki indicates that entry is not the only factor defining the cell and tissue tropism of these viruses. Thus, UGV-1 and HISV-1 reach very high titers in I/1Ki [22] though our results showed low efficiency of cell entry mediated by these virus GPs. Horizontal transmission of reptarenavirus infection has been documented [21], suggesting potential aerosol transmission. Our results showed that most reptarenavirus GPs could mediate entry into boa (V/5Lu) and human (A549) lung cells (Figure 4 and Table 1). The lower VSV replication efficiency in V/5Lu (Figure 3) could have contributed to the observed lower entry rate in this cell line. Additionally, the cell line might not represent the optimal target cells of reptarenaviruses. In BIBD, reptarenavirus infection is often associated with CNS signs, and we originally identified TSMV-2 in the brain of a snake with multiple reptarenavirus L and S segments in the blood [23]. Curiously, the boa brain cell line (V/4Br) proved to be poorly permissive for pseudotypes bearing arenavirus GPs, especially for those of UHV-1, HISV-1, CASV-1, TSMV-2, and S5-like (Figure 4 and Table 1). While this finding is rather surprising, it could reflect the fact that the reptilian cell lines included in the study represent quite homogenic cell populations that derive from spontaneously immortalized cultures generated from homogenates of different tissues. Given the limitation of our study, the results do not rule out the possibility of efficient viral replication in the brain nor in this particular cell line, since reptarenavirus IBs are generally frequent in neurons in the brain of boas [13,14,15,23]. The reptarenavirus GPs differentially mediated entry into boa (V/1Liv) and python (VII/1Liv) liver cells; while the boa cells were rather permissive, the python cells appeared to be non-permissive. In snakes with BIBD, the liver cells often exhibit abundant IBs [11,13,15,61], and thus the result could indicate a species tropism. However, as discussed above, several other factors could contribute to this observation. When characterizing HISV-1 we speculated that cell-to-cell transmission plays a role in its life cycle, due to observed alterations at the plasma membrane of HISV-1 infected cells [22]. Cell-to-cell transmission, reported for several viruses [62,63], would also help to explain some of the peculiar results (e.g., low entry efficiency in reptilian cell lines known to support reptarenavirus infection) of the present study using pseudotyped VSV particles.

Mammarenavirus GPs efficiently mediated entry into both mammalian and reptilian cell lines. One could speculate that the entry of mammarenaviruses into snake cells relies on receptor homologies between reptiles and mammals. Basic Local Alignment Search Tool (BLAST) search at NCBI shows that the amino acid identity between human and python (the genes for *B. constrictor* are not available) α-DG is approximately 74%, while the identity between human and python TfR1 is 51%. It is plausible that mammarenavirus entry into snake cells may occur via the corresponding receptor orthologues. JUNV entry efficiency varies between the specific TfR1 species [64], being highest with the TfR1 of JUNV’s reservoir host (*Calomys musculinus*) [36]. JUNV enters human cells through TfR1, and e.g., the TfR1 orthologue of the cat (*Felis catus*) can mediate JUNV entry while the TfR1 orthologues of mouse and rat do not support its entry [36]. Several OW mammarenaviruses use α-DG as a main receptor for cell entry [38]. However, these OW mammarenaviruses can use additional cell surface receptors and cofactors for efficient entry [65,66]. Both LCMV and JUNV GPs showed similar preferences for mammalian and reptilian cell cultures in our study. The fact that we used native cell lines as opposed to cell lines overexpressing TfR1 and α-DG could be compatible with both TfR1 and α-DG dependent and independent entry. The observed differences in the entry efficiencies of the tested arenavirus GPs provides a good starting point for future studies addressing the receptor preference of reptarenaviruses.

## 5. Conclusions

Herein, we studied the entry abilities of arenaviruses in a spectrum of cultured mammalian and reptilian cell lines. We think that our results are indicative of receptor abundance in the tested cell lines, and thus provide preliminary data for further studies of receptor usage and cell tropism with isolated viruses. The observed differences in the “GP-driven” tropism need to be revisited using isolated reptarena- and hartmaniviruses, since factors other than entry significantly contribute to the tissue and cell tropism. The large swarm of L and S segments in BIBD-infected snakes may have an impact on the receptor-induced restriction of tissue tropism. The python liver cell line, VII/1Liv, which showed to be rather impermeable to the VSV pseudotypes with arenavirus GPs, could serve in future transfection studies to assess the receptor usage of reptarenaviruses. The findings that reptarenavirus GPs efficiently mediate entry into mammalian cell lines and mammarenavirus GPs to reptilian cell lines could indicate an underlying cross-species transmission potential of arenaviruses; however, our earlier study showed mammalian body temperature to be suboptimal for reptarenavirus replication [48].

## Figures and Tables

**Table 1:** Infectivity of the pseudotyped viruses in mammalian and reptilian cell lines. The infectivity values are the average of quadruplicates and expressed in 1000 FFFU/mL. For each virus, light red highlights the cell line with the highest and light blue the cell line(s) with the lowest infectivity.

	Mammalian Cells						Reptilian Cells					
Virus Type	Vero E6	BHK-21	SK-N-NH	CHO wt	HEK293FT	A549	I/1Ki	V/2Hz	V/5Lu	V/4Br	V/1Liv	VII/2Liv
UHV-1	305.9	905.7	19.5	1112.0	748.3	290.6	74.4	230.4	0.2	0.2	4.5	1.2
UGV-1	539.7	1823.4	50.5	2080.0	1297.0	337.2	175.5	326.3	1.8	2.8	5.4	0.4
UHV-2	617.1	2052.5	55.3	2346.1	2238.7	264.2	1421.6	373.8	2.7	1.8	8.4	1.4
GGV-1	1002.7	3800.7	106.1	6567.7	2700.5	357.8	336.6	480.3	4.0	2.1	8.3	0.3
CASV-1	11.9	107.4	2.1	96.0	2819.3	28.1	352.6	517.6	3.8	0.2	0.1	0.5
ABV-1	667.0	1208.6	27.6	2102.9	1448.9	226.9	283.1	463.0	0.8	4.8	11.5	0.8
ABV-2	869.1	2071.6	90.8	3016.5	1485.3	305.0	218.4	367.6	7.3	2.6	13.1	1.0
TSMV-2	304.9	684.7	79.5	1092.6	1223.6	239.7	69.7	97.3	1.2	0.4	1.9	0.1
S-5	543.8	1389.4	65.6	1909.9	1669.5	303.6	596.8	184.9	7.0	0.2	7.7	0.5
HISV-1	512.4	1655.4	47.3	2309.3	1083.5	338.5	148.2	282.6	1.5	0.5	8.6	0.3
LCMV	439.7	1119.4	66.8	1515.3	1738.0	251.8	158.8	320.8	2.7	2.3	12.3	0.4
JUNV	536.9	1284.5	81.1	1994.6	1570.6	190.7	220.1	451.1	3.1	0.9	9.6	0.1
VSV-G	23000.0	10048.6	514.2	6698.9	7056.7	18668.0	7906.8	1755.5	164.2	1254.9	212.0	628.9
